# Supplementary material for: Single-Cell Radiation Response Scoring with the Deep Learning Algorithm CeCILE 2.0
Source: Cells. 2023 Dec 7;12(24):2782. doi: 10.3390/cells12242782 (PMC10742313; doi:10.3390/cells12242782)
Supplement: Supplementary file 1 [file cells-12-02782-s001.zip › cells-2731089-supplementary.pdf]

Supplementary material for the manuscript:

Single-Cell Radiation Response Scoring with the Deep Learning Algorithm CeCILE 2.0

Sarah Rudigkeit and Judith Reindl

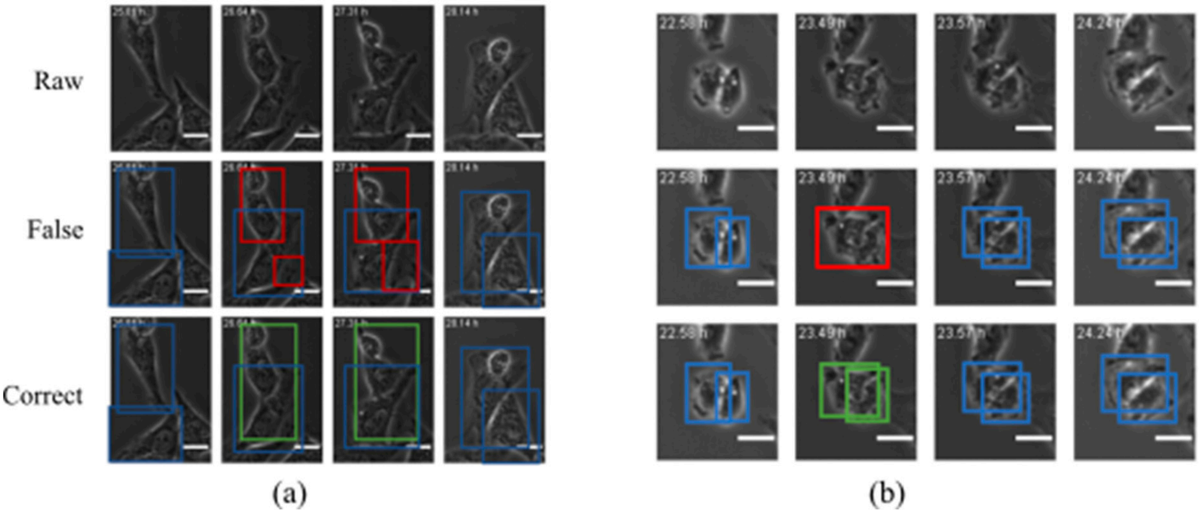

Supplementary Figure S1: (a) Two cells moving on top of each other and (b) two cells close to each other. The first row shows the raw phase-contrast images of the scenes. In the second and third rows, the cells are annotated with bounding boxes. In the second row, the scene is annotated as CeCILE would incorrectly predict this scene, and in the third row, the scene is presented as this scene would be correctly annotated. The red bounding boxes indicate incorrect annotations and the green bounding boxes indicate correct annotations. The scale bar indicates a distance of 20 µm.

Supplementary Table S1: List of the data used for CeCILE 2.0

	# Frames	Intervals	Cell line	#liv	#round	#div	#dead	Total	Substrate	Coating	Irr
1	11	100 min	CHO-K1	1342	238	5	23	1608	Glass	Gelatine	H^+ 4Gy
2	10	100 min	CHO-K1	2456	473	11	75	3015	Glass	Gelatine	H^+ 4Gy
3	11	100 min	CHO-K1	208	167	1	40	416	Glass	Gelatine	No
4	11	100 min	CHO-K1	2526	346	9	33	2914	Glass	Gelatine	H^+ 4Gy
5	10	100 min	CHO-K1	1608	373	8	122	2111	Glass	Gelatine	H^+ 4Gy
6	10	100 min	CHO-K1	2209	352	9	44	2614	Glass	Gelatine	H^+ 4Gy
7	11	100 min	CHO-K1	403	269	3	25	700	Glass	Gelatine	No
8	11	100 min	CHO-K1	365	167	2	18	552	Glass	Gelatine	H^+ 4Gy
9	70	20 min	Hela	1135	209	3	1515	2862	Scintillator	Celltak	C^{6+} 1Gy
10	70	20 min	Hela	2093	145	4	1669	3911	Scintillator	Celltak	C^{6+} 2Gy
11	42	20min	Hela	2302	100	13	1573	3988	Scintillator	Celltak	C^{6+} 4Gy
12	42	20min	Hela	1154	110	15	1331	2610	Scintillator	Celltak	No
13	7	100 min	CHO-K1	552	102	8	24	686	Glass	Gelatine	H^+ 4Gy

14	12	Random	CHO-K1	967	101	13	91	1172	Plastic	No	No
15	21	Random	LN229	2108	119	38	82	2347	Plastic	Gelatine	No
16	17	Random	LN229	1412	93	41	147	1693	Plastic	Gelatine	No
17	13	Random	LN229	738	65	17	44	864	Plastic	Gelatine	No
18	4	Random	LN229	244	51	7	25	327	Plastic	Gelatine	No
19	20	Random	Hela	901	29	28	61	1019	Plastic	No	No
20	20	Random	Hela	982	35	25	10	1145	Plastic	No	No

Supplementary Table S2: Details for the videos used for tracking.

	# Frames	Intervals	Cell line	#liv	#round	#div	#dead	Total	Substrate	Coating	Irr
1	457	5 min	CHO-K1	24042	2447	106	540	32025	Glass	No	X-rays 3Gy
2	341	5 min	CHO-K1	23574	1159	117	2704	27554	Glass	No	No
